# Commentary on Ziermans T. et al. “Call to Action on Social Cognition Measures”

**DOI:** 10.1016/j.scog.2025.100413

**Published:** 2025-12-19

**Authors:** Ruben C. Gur, Raquel E. Gur

**Affiliations:** a Department of Psychiatry, University of Pennsylvania, and the Penn-CHOP Lifespan Brain Institute (LiBI), Philadelphia, PA, United States

It is difficult to overstate the importance of Ziermans et al.'s Call. With the increased realization that psychiatric disorders are behavioral manifestations of the dysfunction of neural systems regulating all aspects of behavior, the past emphasis on cognition and executive functions has left a gaping hole in understanding social cognition and its deficits associated with psychopathology. The Call “to develop psychometrically sound and ecologically valid SC tools” (p. 8) is sound and timely, with equal emphasis on psychometric rigor and ecological validity, the latter implying construction and validation of tools that would incorporate racial and ethnic diversity across cultures and ethnicities. Importantly in our view, these measures should be computerized and implementable on any web enabled computer or a hand-held device so that they can be administered not only by highly trained professionals in major research centers but also in any clinic or the participant's home. Such tools can also be applied in neurodevelopmental cohorts with appropriate adjustments to the younger age groups. Such work will contribute to understanding the increased risk for schizophrenia spectrum disorders in other neurodevelopmental disorders that emerge in childhood (Gur, 2025). Incorporating wearables and ecological momentary assessment (EMA) could further enhance our ability to measure SC parameters with increased spatio-temporal resolution.

We admittedly took some pride in the finding of the prominence of the ER-40. It is noteworthy that the ER-40 was conceived and developed with the same principles articulated in the “Call”. The face stimuli were carefully constructed and validated in diverse populations (e.g., Pinkham et al (Pinkham et al., 2008).) and represent males and females of diverse races and ethnicities across the age range. Perhaps this effort paid off by increasing the acceptability of the tool.

In some ways, the task for developing such tools for SC is easier than for other cognitive measures that require language. Stimuli for SC measures can be found in the public domain (e.g., CREMA-D (Cao et al., 2014)) and could be leveraged to expand the range of SC constructs tapped by specific tests. For example, in addition to emotion identification measured with the ER-40, a parallel task could be developed with available technology that would measure the ability to express emotions. Furthermore, while the face is a reasonable place to start investigating social cognition since so much of our social interactions involve faces, other aspects of social cognition, such as voice and body posture, need to be incorporated into future measures.

While seconding the Call for more work on SC, we would like to add a tweet in support of examining other relatively neglected aspects of behavior that are highly relevant to psychopathology and have well-defined neural substrates. Specifically, the reward system has been implicated in schizophrenia and other disorders, and thus far there is dearth of validated measures. Studies that have examined effort, delay and risk discounting have found associations with negative symptoms in schizophrenia (Prettyman et al., 2021), and more detailed evaluation of motivational aspects of behavior could yield further insights on the pathophysiology of mental disorders.

## CRediT authorship contribution statement

**Ruben C. Gur:** Conceptualization, Writing – original draft, Writing – review & editing. **Raquel E. Gur:** Conceptualization, Writing – original draft, Writing – review & editing.

## Declaration of competing interest

The authors declare no competing interests.
